# From Head and Neck Tumour and Lymph Node Segmentation to Survival Prediction on PET/CT: An End-to-End Framework Featuring Uncertainty, Fairness, and Multi-Region Multi-Modal Radiomics

**DOI:** 10.3390/cancers15071932

**Published:** 2023-03-23

**Authors:** Zohaib Salahuddin, Yi Chen, Xian Zhong, Henry C. Woodruff, Nastaran Mohammadian Rad, Shruti Atul Mali, Philippe Lambin

**Affiliations:** 1 The D-Lab, Department of Precision Medicine, GROW—School for Oncology and Reproduction, Maastricht University, 6200 MD Maastricht, The Netherlands; 2 Key Laboratory of Intelligent Medical Image Analysis and Precise Diagnosis, College of Computer Science and Technology, Guizhou University, Guiyang 550025, China; 3 Department of Medical Ultrasonics, Institute of Diagnostic and Interventional Ultrasound, The First Affiliated Hospital of Sun Yat-sen University, Guangzhou 510080, China; 4 Department of Radiology and Nuclear Medicine, GROW—School for Oncology and Reproduction, Maastricht University Medical Center+, 6229 HX Maastricht, The Netherlands

**Keywords:** head and neck cancer, segmentation, uncertainty estimation, explainability, CT radiomics, PET radiomics, fair artificial intelligence

## Abstract

**Simple Summary:**

Automatic delineation and detection of the primary tumour and lymph nodes using PET and CT in head and neck cancer can be helpful for diagnosis, prognosis, and monitoring the disease. However, these algorithms can suffer from silent failures, limiting their trust. In this research work, we estimate the confidence of the predicted segmentation and use it to reduce the number of false predictions. We also investigate the prognostic potential of quantitative image features extracted from the primary tumour and lymph nodes. We combine these features with clinical characteristics to predict recurrence-free survival and stratify patients into three groups of low, medium, and high-risk patients. We gain insight into the decision-making process of the model using explainability methods and correlate it to clinical knowledge. We also evaluate if the models are impacted by different biases. Our proposed framework can aid clinicians in the detection of head and neck cancer and patient risk stratification.

**Abstract:**

Automatic delineation and detection of the primary tumour (GTVp) and lymph nodes (GTVn) using PET and CT in head and neck cancer and recurrence-free survival prediction can be useful for diagnosis and patient risk stratification. We used data from nine different centres, with 524 and 359 cases used for training and testing, respectively. We utilised posterior sampling of the weight space in the proposed segmentation model to estimate the uncertainty for false positive reduction. We explored the prognostic potential of radiomics features extracted from the predicted GTVp and GTVn in PET and CT for recurrence-free survival prediction and used SHAP analysis for explainability. We evaluated the bias of models with respect to age, gender, chemotherapy, HPV status, and lesion size. We achieved an aggregate Dice score of 0.774 and 0.760 on the test set for GTVp and GTVn, respectively. We observed a per image false positive reduction of 19.5% and 7.14% using the uncertainty threshold for GTVp and GTVn, respectively. Radiomics features extracted from GTVn in PET and from both GTVp and GTVn in CT are the most prognostic, and our model achieves a C-index of 0.672 on the test set. Our framework incorporates uncertainty estimation, fairness, and explainability, demonstrating the potential for accurate detection and risk stratification.

## 1. Introduction

Head and neck (H&N) cancer is the seventh most common cancer worldwide with more than 0.66 million new cases and 0.3 million deaths every year [1]. The incidence rate of H&N cancer is increasing worldwide, and the five-year survival rate for H&N cancer varies from 85.1% in localized cancer cases to 40.1% in distant cancer cases [2,3]. Research has shown a significant association between surgical delay and increased mortality rates in various populations. Patients with stage I or II cancer can achieve at least a 70% five-year survival rate if they receive appropriate treatment [4]. Early diagnosis and staging are important for improving the prognosis of H&N cancer. Computed tomography (CT) and 18F-fluorodeoxyglucose (FDG) positron-emission tomography (PET) are the most commonly used imaging modalities for the initial diagnosis, staging, and follow-up of H&N cancers, as they provide synergistic information related to metabolism and morphology [5,6,7]. With an increasing population, especially in developing countries, an increasing number of images acquired, and an increasing incidence rate for H&N cancer, there is a need to develop automatic segmentation and detection tools for H&N cancer to aid clinicians and reduce their workload. Automatic segmentation models cannot only help clinicians in tumour detection and delineation but also reduce inter- and intra-observer variability [8].

Convolutional neural networks (CNNs), a type of deep learning algorithm adapted to accept images as inputs, are becoming increasingly popular for medical image segmentation tasks. The U-Net architecture is one of the widely used CNNs for medical image segmentation [9,10]. In recent years, U-Net has been used as the backbone for head and neck segmentation [11,12]. No-new-U-Net (nnU-Net) has shown state-of-the-art performance in many medical image segmentation tasks [13]. The nnU-Net has streamlined many design choices related to pre-processing, network architecture, and hyperparameter selection. These design choices in nnU-Net are configured automatically based on the model hyperparameters. However, the success of nnU-Net and other segmentation methods in achieving high scores on segmentation tasks does not ensure the reliability of the segmentation results, as they can fail silently without any notice [14]. This problem is particularly acute when a lot of heterogeneity is present in the medical data. Incorporating an uncertainty estimation can help quantify the reliability and robustness of the segmentation outcomes, as there is a positive correlation between uncertainty and segmentation error [15]. Therefore, an uncertainty estimation is critical for the clinical deployment of the segmentation models [16]. 

Radiomics is a quantitative image analysis technique that can be divided into handcrafted radiomics (HCR) and deep learning (DL). Handcrafted radiomics extracts imaging features from radiographic medical images and correlates them with clinical and biological information using machine learning methods [17,18,19]. Deep learning involves training artificial neural networks to learn representative features for outcome prediction from amounts of data, and deep learning-based models have been developed to predict progression-free survival for head and neck squamous cell carcinoma patients using clinical and PET/CT imaging data [20,21,22]. Several studies have shown that radiomics in CT has the potential to improve the prediction of the prognosis of H&N cancer [23,24,25]. Some studies have also investigated the use of radiomics in both CT and PET for survival analysis for H&N cancer [26,27]. While these studies investigate the prognostic potential of CT and PET radiomics based on primary tumour delineation, there is a need to quantify the prognostic potential of radiomics features extracted from different regions of interest (ROI) such as the primary tumour and lymph nodes. Furthermore, it is also necessary to evaluate the predictive potential of radiomics features from different imaging modalities of CT and PET. The radiomics features can be extracted from the primary tumour and lymph nodes independently from both PET and CT images. 

Handcrafted radiomics and deep learning has the potential to revolutionize the field of radiology. However, AI algorithms can lead to biased tools that replicate and amplify health inequalities between different cohorts, such as gender, age, and income [28,29]. The fairness principle of FUTURE-AI emphasises that AI algorithms should be impartial to individual and group differences, and they should demonstrate similar performance irrespective of the disparities [30]. An investigation into the fairness of the algorithm for abnormalities detection in chest X-rays revealed that the algorithm shows biased performance with respect to sex, age, and ethnicity [31]. A study on cardiac segmentation performance with respect to fairness revealed that a dataset which is balanced with respect to gender but imbalanced with respect to ethnicity does not perform consistently with respect to ethnicity [32]. Therefore, it is important to evaluate the performance of the algorithm with respect to possible biases to identify if there are any significant differences in the performance of the algorithm. 

In this paper, we propose an end-to-end framework for survival analysis based on the automatic detection and segmentation of tumours and lymph nodes as well as uncertainty estimation. The key contributions of this work are as follows:

- We evaluated a 3D segmentation framework for primary tumour and lymph node segmentation.
- We implemented a method for uncertainty estimation to calculate the model confidence for the primary tumour and lymph nodes segmentation to minimise the risk of the model failing silently. We applied the uncertainty score for the false positive reduction in lymph nodes and tumours.
- We extracted handcrafted radiomics features both from the primary tumour and lymph nodes, separately from CT and PET images, and investigated their prognostic potential. We explored different combinations of these regions of interest in these two modalities to guide future research.
- We evaluated the performances of the segmentation model and the radiomics model for fairness with respect to relevant clinical characteristics such as age, gender, HPV status, and chemotherapy status, as well as lesion size.

## 2. Materials and Methods

### 2.1. Dataset

Our work is based on the HECKTOR 2022 challenge dataset (https://hecktor.grand-challenge.org/Timeline/, accessed on 20 August 2022), which includes 883 H&N cancer patients from 9 different centres [33,34]. The training set consisted of 524 patients acquired from 7 centres, and the test set consisted of 359 patients from 3 centres. All patients have histologically proven oropharyngeal H&N cancer and have been treated with radiotherapy and/or chemotherapy. For all patients, PET/CT scans were acquired, and some patient information such as institution, age, sex, weight, tobacco, alcohol use, performance status, human papillomavirus (HPV) status, and treatment (radiation only or adjuvant chemo and/or surgery) were collected.

For the segmentation task, the annotation of primary gross tumour volume (GTVt) and nodal gross tumour volume (GTVn) of all 524 patients are provided as ground-truth masks in the training dataset. For the survival prediction task, recurrence-free survival (RFS), including time-to-event in days and censoring, is provided as ground truth labels of 489 patients in the training set. Due to the availability of data, 359 patients and 339 patients in the test set were used for the segmentation task and the survival prediction task, respectively.

### 2.2. Segmentation

The multicentric data contained CT and PET images with varying resolutions. The median resolution of CT images in the training dataset was 0.98 × 0.98 ×3.27 ${\mathrm{mm}}^{3}$. We resampled the PET and CT to a common resolution of 1 × 1 × 3 ${\mathrm{mm}}^{3}$. The CT intensity values were clipped at 0.5 and 99.5 percentiles. We applied z-score normalization and min-max normalization for CT and PET images respectively in accordance with nnUNet guidelines [13]. We applied Otsu thresholding to the PET scan and found the first four axial slices containing the brain region starting from the top. We found the centre $I_{c-axial}$ of the thresholded region in the axial plane. We cropped a region of 260 × 260 × 104 voxels (or 260 mm × 260 mm × 312 mm) centred around $I_{c-axial}$, starting from the top of the detection brain region. This cropping step reduced the image size, which decreased the computation cost and allowed the model to focus on the relevant region for head and neck cancer detection. The size of 260 × 260 × 104 voxels was selected after ensuring that all the ground truth tumours and lymph nodes were encapsulated within this region and no loss of information had occurred.

**Figure 1:**
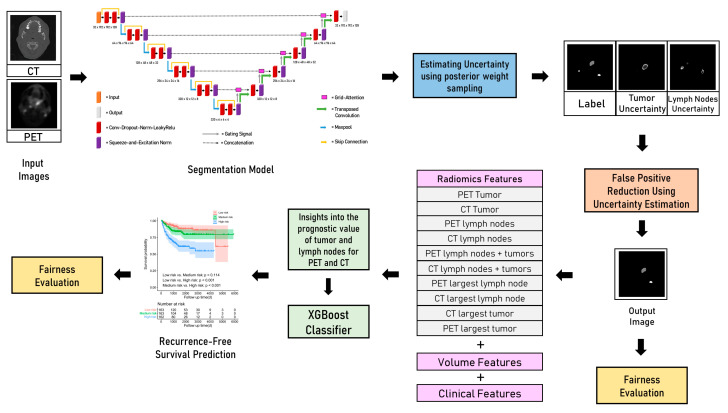
End-to-end primary tumour and lymph nodes segmentation and survival prediction framework with segmentation uncertainty estimation, exploration of the prognostic potential of lymph nodes, tumour regions of interest for radiomics feature extraction for survival prediction, and fairness evaluation.

Figure 1 summarizes all the components present in our proposed framework. The proposed segmentation model has been shown in the first step. We modified the 3D nnU-Net [35] to include residual skip connections [36], squeeze-and-excitation channel-wise attention mechanisms (SE) at each layer [37], and grid attention gates (GA) at each skip connection [38]. The SE layer aids in learning useful features by fusing spatial and channel-wise features. Grid attention gates can aid in reducing the number of false positives by allowing the model to focus on the relevant parts of the image. The patch size for training was set at 192× 192 × 64 voxels. Random rotation, random scaling, random elastic deformation, mirroring, the addition of gaussian noise, and gamma correction were applied to each patch for augmentation. Multi-class Dice loss [39] and cross-entropy loss were used to train the model with deep supervision to allow the loss to be calculated at each stage of the decoder. We trained the model for 1200 epochs to allow for convergence and used a stochastic gradient descent (SGD) optimizer for training with a learning rate of 0.01 and a momentum of 0.9. We performed fivefold cross-validation on the training dataset and use the ensemble of the five models from the fivefold for prediction on the test set.

### 2.3. Uncertainty Estimation

We incorporated uncertainty estimation in the segmentation model to avoid silent failures and estimated the model’s confidence for the predicted segmentation mask. We employed posterior sampling of the weight space to estimate uncertainty by saving posterior models at appropriate moments during stochastic gradient descent (SGD) training as proposed in [15]. When the number of epochs increases, the polynomial learning rate results in model convergence as the learning rate diminishes to zero. When trained for long epochs using SGD, the saved weights oscillate around the local minimum after a sufficient number of epochs have elapsed. Let the training data set consisting of N training images be denoted by $D={\left\{\left(x_{i},\;y_{i}\right)\right\}}_{i=1}^{N}$, where $x_{i}$ represents the training images and $y_{i}$ represents the corresponding segmentation masks.

For a test image $\bar{x}$ and predicted segmentation mask $\bar{y}$, the prediction can be given by:

$$p(\bar{y}|\bar{x},\;D)=\frac{1}{n}\cdot \overset{n}{\underset{i=1}{\sum }}p(\bar{y}|\bar{x},\;w_{i})$$

$w_{i}$ represents the saved checkpoint weights when the SGD training stabilises and γ training epochs have elapsed. The predicted posterior $p(\bar{y}|\bar{x},D)$ is the average of the prediction of N models with weights $w_{i}$ where i=1 to N.

We utilised the multi-model posterior weight sampling around multiple local optima as proposed in [15] to make use of the diversity of weight samples. We used a cyclic learning rate for exploring multiple local optima. The total training budget $T_{max}$ is set at 1200 to allow for convergence within each training cycle. $T_{max}$ is divided into $N_{cycles}=3$. Each cycle consist consists of $T_{cycle}=\frac{T_{max}\;}{N_{cycles}}=400$ epochs. We kept the gradient-step constant after γ = 80% of the training budget in the cycle $T_{cycle}$ has elapsed. At any point in the cycle, the learning rate follows the following equation:

$$lr\;\left(t\right)=0.1,t_{mod}=0$$

$$lr\;\left(t\right)=0.01\;\cdot {\left[1-\frac{min\left(t_{mod},\;320\right)\;}{400}\right]}^{\;0.9}\;,\;t_{mod}>0$$

$t_{mod\;}=t\;\%\;T_{cycle}=t\;\%\;400$ represented the number of epochs elapsed within the cycle. At $t_{mod\;}$= 0, we set a high learning rate of 0.1 for one epoch at the start of the cycle to get out of the local optimum. For $t_{mod\;}$> 0, we employed polynomial learning rate decay within each cycle to reach model convergence. After 320 epochs within each cycle, the gradient step is constant, and we randomly save 10 checkpoints per cycle. 30 checkpoints are saved from 3 different cycles, constituting the multi-model posterior samples in the weight space. We quantified the uncertainty by calculating the variance of the predictions $I_{uncertain}$ made by 30 different models. High values of variance correlate with high uncertainty and demonstrate that the model is unsure about the prediction. Furthermore, the correlation of false positives with high uncertainty can be exploited for reducing false positives per image. The uncertainty density $t_{uncertain}$ of the lesion $l_{i}$ is calculated as follows:

$$t_{uncertain\;}=\frac{1\;}{N}\cdot \sum I_{uncertain}\left(x,y,z\right)\cdot l_{i}\left(x,y,z\right)$$

$I_{uncertain}$(*x*, *y*, *z*) represent the uncertainty value of a pixel at the coordinate (*x*, *y*, *z*) in the image and $l_{i\;}$(*x*, *y*, *z*) represented a binary lesion mask. We set $t_{uncertain}$ at various thresholds to reduce false positives and investigated the impact on both the false positives per image and the sensitivity of detecting tumour and lymph nodes.

### 2.4. Handcrafted Radiomics

The segmentation masks predicted by the model were used for extracting radiomics features. A total of 107 radiomics features were extracted using PyRadiomics including first-order features, shape features, and texture features (Appendix A, Table A1). These radiomics features were extracted from five different ROIs from CT and PET images separately. The bin width for intensity discretisation was set at 25 and 0.5 for CT images and PET images, respectively, to ensure that the bin count is greater than 30 for reproducibility and better performance [40]. All the radiomics features were extracted from 3D region of interests (ROI). CT_all and PET_all represented radiomics features extracted from combined primary tumour and lymph node regions from CT and PET images respectively. CT_only_tumour and PET_only_tumour represented radiomics features extracted from the tumour region only from CT and PET images respectively. The segmentation model can predict multiple tumours and lymph nodes. CT_largest_tumour and PET_largest_tumour represented radiomics features extracted from the largest tumour region from CT and PET images respectively. CT_only_lymph and PET_only_lymph represented radiomics features extracted from the lymph nodes region only from CT and PET images, respectively. CT_largest_lymph and PET_largest_lymph represented radiomics features extracted from the largest lymph node region from CT and PET images, respectively. The radiomics features also included 6 volume-related features i.e., total tumour volume, largest tumour volume, total lymph nodes volume, largest lymph node volume, the total number of lymph nodes and the total number of tumours. All 9 available clinical features, namely, gender, age, weight, chemotherapy, tobacco, alcohol, surgery, HPV status, and performance status, were included as clinical features.

Feature selection is performed in two steps. Univariate analysis is performed to select the top 10 features with the highest C-index in a Cox proportional hazards model in the first step. These features are then aggregated one by one in the order of C-index to find the best combination. A model based on the XGBoost classifier with a regression tree base learner is trained for survival prediction. The ten sets of radiomics features from CT and PET images along with volume and clinical features are explored individually for survival prediction. Feature selection is performed to evaluate the performance of radiomics features extracted from a specific modality and region of interest. All the selected features are aggregated along with clinical features, and feature selection is performed again to form the radiomics_clinical model. Fivefold cross-validation is performed on the training set for hyperparameter tuning and evaluation. The final radiomics_clinical model is tested on the unseen external test dataset. Shapley additive explanations (SHAP) is a post-hoc interpretability method that helps in measuring the importance of each individual feature on the model’s prediction in terms of SHAP values. SHAP global summary plots were used to study the impact of features in the radiomics_clinical model.

### 2.5. Fairness

The performance of artificial intelligence algorithms, in particular deep neural networks, is highly correlated with the quality and distribution of the training data [41]. For equitable AI, we need to ensure that the performance of AI algorithms remained the same when they are applied to various groups of individuals with varying characteristics [30]. The problem of equitable and fair AI is aggravated in the case of unbalanced datasets. We evaluated the performance of the proposed segmentation model and the radiomics_clinical model with respect to age, gender, chemotherapy, and HPV status during fivefold cross-validation.

### 2.6. Evaluation Metrics

#### 2.6.1. Dice Coefficient

Dice coefficient (DSC) measures the spatial overlap between the ground truth mask and the predicted mask. The formula for DSC is as follows:

$$DSC=\frac{\;2\;\left|\hat{I}\;\cap \;I\right|}{\;\left|\hat{I}\right|+\left|\;I\right|}$$

where I refers to the ground truth mask, and $\hat{I}$ refers to the predicted mask.

#### 2.6.2. Aggregated Dice

Aggregated Dice ($DSC_{agg}$) is derived from the aggregated Jaccard index. All the intersections and unions between the ground tumour volumes (GTVs) and their respective predicted volumes are accumulated across all images [33]. The aggregated intersection is divided by the aggregated union. The formula is as follows:

$$DSC_{agg}=\frac{2\;\sum_{i}^{N}\sum_{k}{\hat{I}}_{i,k}I_{i,k}}{\sum_{i}^{N}\sum_{k}({\hat{I}}_{i,k}+I_{i,k})}$$

where $I_{i,k}$ is the ground truth for voxel k and image i, and ${\hat{I}}_{i,k}$ is the prediction. $DSC_{agg}$ of the primary tumour is denoted by $DSC_{agg}$ GTVp, and $DSC_{agg}$ of lymph nodes is denoted by $DSC_{agg}$ GTVn.

#### 2.6.3. Sensitivity

The threshold of Dice coefficient DSC for detection is set at 0.1. If DSC for a lesion was less than 0.1, it was not detected. This was in line with previous studies that considered the non-overlapping nature of 3D lesions [42,43,44]. The ground truth lesions that have DSC > 0.1 with predicted lesions are counted as true positives (TP), and the ground truth lesions with DSC < 0.1 are counted as false negatives (FN). The sensitivity is given by:

$$Sensitivity=\frac{TP}{TP+FN\;}$$

#### 2.6.4. C-Index

The C-index generalises the area under the receiver operator characteristic (ROC) curve by quantifying the model’s ability to provide a good split between the survival curves. C-index is based on the computation of individual patient risk scores while accounting for censored data. This statistical tool provides a global evaluation of the model’s discrimination power.

### 2.7. Statistical Tests

The Mann–Whitney test and Chi-squared test were used to compare the variables between the training and test set. The Mann–Whitney test was used to check for statistically significant differences in Dice score and sensitivity between each subgroup and the total reported statistics. A two-sided permutation test was used to check for statistical significance for C-index for survival prediction and aggregated Dice score. For survival prediction, three risk groups were determined using threshold values at the 33rd and 66th percentile of the calculated risk score. The survival curves were generated using the Kaplan–Meier method. Two log-rank tests were performed to determine the significance of the split of the low- vs. the medium-risk groups, and the medium- vs. the high-risk groups.

## 3. Results

### 3.1. Patient Characteristics

The clinical characteristics of both the training and test datasets are shown in Table 1. In the training dataset, the gender ratio was 95/429 (female/male), and the median age was 61.0 (IQR 54.0–67.0) years. In the test dataset, the gender ratio was 63/296 (female/male), and the median age was 59.0 (IQR 53.2–66.0) years. There were no significant differences in gender, weight, and chemotherapy between the training and test datasets (all *p* > 0.05). There is a significant difference in age between the training and test datasets (*p* = 0.024). We evaluated the influence of age on performance by dividing age into four equal quartiles: 0 to 50, 50 to 60, 60 to 70, and more than 70 years. Similarly, we also evaluated the performance of the segmentation with respect to lesion size by dividing the size into four equal quartiles: 0 to 4.03 cm^3^ (small), 4.03 to 7.70 cm^3^ (medium), 7.70 to 17.3 cm^3^ (large), and over 17.3 cm^3^ (extra large).

### 3.2. Segmentation

The segmentation results are reported in Table 2. For fivefold cross-validation, the model achieved a mean sensitivity of 0.964 for tumours and 0.878 for lymph nodes, a mean Dice score of 0.725 for tumours and 0.658 for lymph nodes, and a mean $DSC_{agg}$ GTVp of 0.808 and a mean $DSC_{agg}$ GTVn of 0.780. On the unseen external dataset, the segmentation model achieved $DSC_{agg}$ GTVp of 0.774 and $DSC_{agg}$ GTVn of 0.760.

Dice score DSC, aggregated Dice score $DSC_{agg}$, and detection sensitivity for tumours and lymph nodes showed no statistically significant difference in performance in different age groups and different genders when compared with the overall performance of the segmentation model (all *p* > 0.05). There was a statistically significant difference in performance for $DSC_{agg}$ and sensitivity for patients who have not had chemotherapy (*p* < 0.05). The detection sensitivity was significantly higher for HPV-negative patients (*p* < 0.05). DSC and $DSC_{agg}$ were significantly lower for small tumours and lymph nodes and higher when tumours and lymph nodes were larger when compared to the overall performance (*p* < 0.05).

### 3.3. Uncertainty Estimation

**Figure 2:**
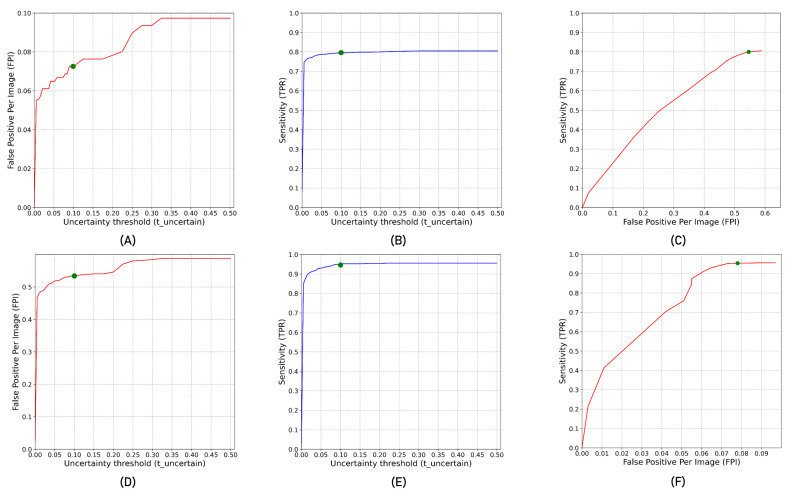
(**A**,**B**) show false positive per image (FPI) and sensitivity/true positive rate (TPR) for tumours at different uncertainty density thresholds $t_{uncertain}$. (**D**,**E**) show FPI and TPR for lymph nodes at different $t_{uncertain}$. (**C**,**F**) FPI vs. TPR curve at different $t_{uncertain}\;$ for tumours and lymph nodes, respectively.

Figure 2 shows the impact of varying $t_{uncertain}$ from 0 to 0.5 in steps of 0.025 on false positive per image and sensitivity. As the threshold for detecting false positives using $t_{uncertain}$ decreases, false positives decrease with a decrease in sensitivity. Figure 2A,B show the impact of per image false positive reduction and sensitivity with $t_{uncertain}$ for the primary tumour. Similarly, Figure 2D,E show the impact of per image false positive reduction and sensitivity with $t_{uncertain}$ for the lymph nodes. Figure 2C,F show the plot of per image false positives and the corresponding sensitivity for primary tumour and lymph nodes, respectively. The green point in Figure 2 showed the ideal operating point that corresponds to $t_{uncertain}=0.1$. At this operating point, there is a per image false positive detection reduction of 7.14% and 19.5% at 0.62% and 0.21% decrease in detection sensitivity for lymph nodes and tumours, respectively.

**Figure 3:**
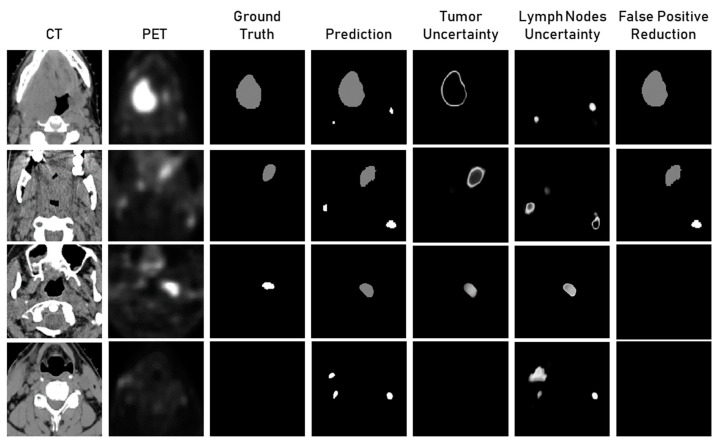
Qualitative analysis of the lymph nodes and tumour segmentation results, tumour and lymph nodes uncertainty maps, and segmentation results after false positive reduction using uncertainty density threshold $t_{uncertain}$.

Figure 3 shows the qualitative results for uncertainty estimation using posterior weight sampling for tumour and lymph node segmentation. The first row in Figure 3 showed that the model wrongly predicted two lymph nodes. The uncertainty of one of these lymph nodes is high and, therefore, $t_{uncertain}$ is high. After applying $t_{uncertain}$ to the threshold, one of the false positives is removed. The uncertainty of the tumour region was high around the boundary. High values of $t_{uncertain}$ were also utilised for the false positive reduction in the second and fourth row of examples in Figure 3. In the third row, the model wrongly predicted a lymph node as a tumour. The uncertainty for both tumour and lymph nodes was high for this region.

### 3.4. Recurrence-Free Survival Prediction

**Figure 4:**
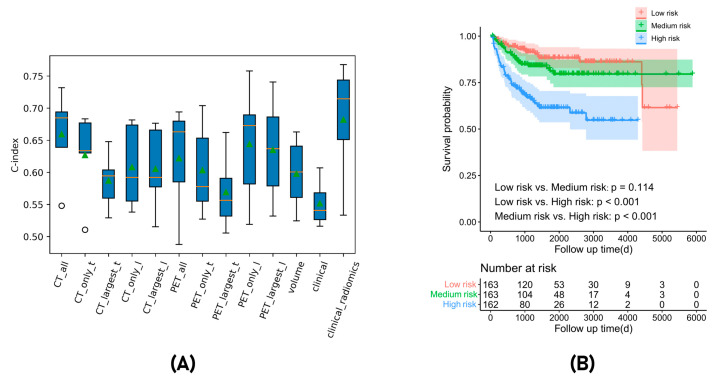
(**A**) Box plot showing the comparative performance of radiomics features extracted from different regions of interest, clinical, volumetric features, and the final radiomics_clinical model. (**B**) Kaplan–Meier survival plots stratified based on the predicted risk score in fivefold cross-validation.

Our results of RFS prediction are reported in Table 3 and Figure 4A. For fivefold cross-validation, the average C-index and standard deviation are reported. We first evaluated the radiomics prediction performance of using different ROIs (all regions including tumour and lymph node, only tumour, only lymph node, largest tumour, and largest lymph node) on PET or CT separately. The results showed that in CT, the radiomics model of all regions performed best with a C-index of 0.659 ± 0.063. In PET, the radiomics model of the largest lymph node performed best with a C-index of 0.644 ± 0.084. Radiomics models based on CT performed better than PET models if only the tumour region is considered. However, radiomics models based on PET performed better than models based on CT if the only lymph node is considered. Radiomics models based on the primary tumour performed similarly to models based on lymph nodes if only CT was considered. However, radiomics models based on lymph nodes performed better than models based on the primary tumour if only PET is considered. The radiomics_clinical model, which combined the radiomics features from different regions in both imaging modality as well as the volume and clinical features, led to the highest C-index of 0.682 ± 0.083 in fivefold cross-validation. Figure 4B showed a Kaplan–Meier survival plot in which all patients were stratified by the predicted risk scores in fivefold cross-validation. The high-risk group showed significantly worse survival than the low-risk group (*p* < 0.001) while the low-risk group and median-risk group did not show a significant difference (*p* = 0.114). The model showed a C-index of 0.672 in the test set.

The C-indexes for different subgroups for fairness evaluations of the radiomics_clinical model during fivefold cross-validation are shown in Table 4. There was no statistically significant difference in model performance for different subgroups of age, gender and lymph node size (all *p* > 0.05) compared with the overall population. However, the model showed significantly lower performance in subgroups without chemotherapy (C-index: 0.555, *p* = 0.022), without HPV infection (C-index: 0.551, *p* = 0.006), and the large tumour size subgroup (C-index: 0.561, *p* = 0.037).

**Figure 5:**
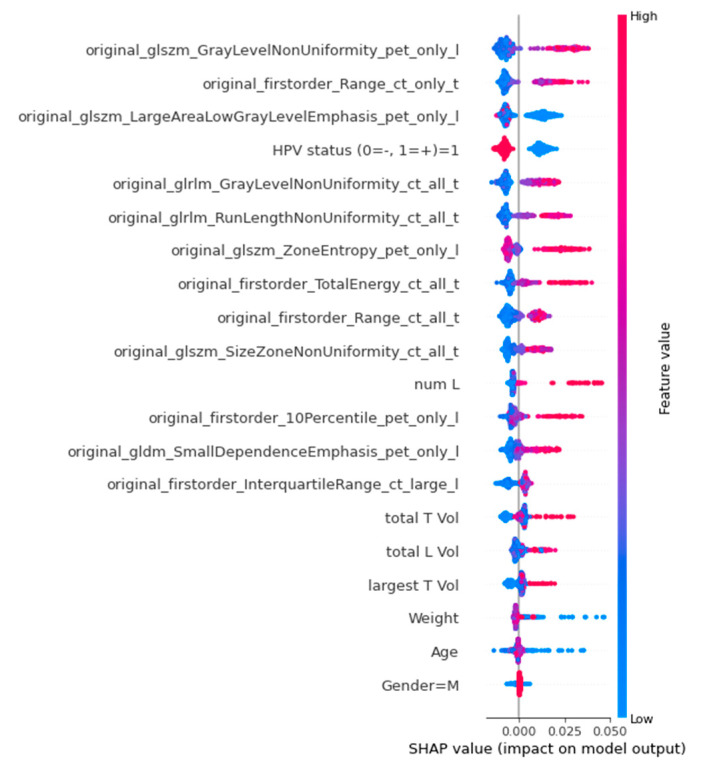
Global SHAP summary plots demonstrate the impact of all the features in radiomics_clinical model in terms of SHAP values. A higher SHAP value on the x-axis corresponds to a positive impact on the model’s output.

A global SHAP summary plot (Figure 5) identified glszm_GrayLevelNonUniformity from lymph nodes in PET, firstorder_Range from tumours in CT, glszm_largeAreaLowGrayLevelEmphasis from lymph nodes in PET, HPV status, and glrlm_GrayLevelNonUniformity from both tumour and lymph nodes in CT as the top five most important features for RFS prediction. Among these features, glszm_GrayLevelNonUniformity from lymph nodes in PET, firstorder_Range from tumours in CT, and glrlm_GrayLevelNonUniformity from both tumour and lymph nodes in CT had a similar trend that a higher feature value resulted in a higher positive SHAP value and higher risk score. While glszm_largeAreaLowGrayLevelEmphasis from lymph nodes in PET and HPV status had a negative trend in which a higher feature value resulted in a lower negative SHAP value and lower risk score. The definitions of all the radiomics features used in radiomics_clinical model are presented in Appendix A, Table A2.

## 4. Discussion

In this study, our proposed segmentation model could detect tumours and lymph nodes during fivefold cross-validation with a detection rate of 96.4% and 87.8%, respectively. The aggregated Dice scores for tumours and lymph nodes were found to be 0.774 and 0.760 in the unseen external test set, respectively. We utilised posterior weight space sampling for uncertainty estimation of tumour and lymph node segmentation to avoid silent failures and measure model confidence. By applying an uncertainty density threshold, there was a reduction of 7.14% in false positives per image for lymph nodes and 19.5% for tumours, with only a minimal decrease in sensitivity. The combination of radiomics features from multiple regions in multi-modality imaging (PET and CT), volume features, and clinical features allowed us to achieve a C-index of 0.672 on the unseen test dataset. The evaluation of fairness showed that the factors such as chemotherapy, HPV status, lesion size, and lymph node size can have an impact on the segmentation results and on the survival prediction.

The reliability of segmentation models could be increased by incorporating an uncertainty estimation to highlight the uncertain areas of segmentation. This can help in identifying false positives as well as warn clinicians. The qualitative analysis of the predicted segmentation along with uncertainty maps for tumours and lymph nodes showed that false positives of lymph nodes have high uncertainty associated with them. Furthermore, the correct segmentations have high uncertainty around the boundary, which correlates well with the fact that the inter-observer variability is also high around the margins of the lesion. We also observed that when the model mistakes the lymph node as a tumour, the uncertainty for both the lymph node and the tumour was high. This showed that uncertainty estimation could also be used for identifying when the model is unsure about the type of lesion. We could obtain a significant decrease in the false positive rate with minimal loss of sensitivity showing that uncertainty is highly correlated with false positives.

The prediction of outcomes was crucial for patients with head and neck (H&N) cancer as it helps clinicians in making informed therapeutic decisions and improving treatment outcomes. Many studies have explored the use of handcrafted radiomics and deep learning in predicting RFS in H&N cancer. Meng et al. [45] developed an outcome prediction model that combines the Deep Multi-task Survival model (DeepMTS), radiomics features, and clinical indicators, achieving a C-index of 0.681 in the test set. Rebaud et al. [46] proposed a binary-weighted radiomics model that resulted in a C-index of 0.682. Wang et al. [47] introduced a prediction framework that integrates the predicted risk scores from separate clinical feature models, PET radiomics models, and CT radiomics models, achieving a C-index of 0.673. In comparison, our focus was mainly on the radiomics prediction performance in different ROIs and modalities. The results showed that the radiomics models based on CT perform better when only the primary tumour region is considered, while radiomics models based on PET perform better when only the lymph node is considered. These results are valuable for further radiomics outcome prediction research, suggesting that the tumour in CT and the lymph node in PET might provide more prognostic information. The results are consistent with Carvalho’s research [48] which showed higher prognostic value for the radiomics model in metastatic lymph nodes than the primary tumour alone in PET. The reason could be that lymph nodes contain tumour cells that are more aggressive and have therefore more impact on the prognosis in PET. However, lymph node staging is difficult in CT because of low soft-tissue contrast [49], which may be the explanation for the lower performance of lymph nodes in CT. The combination of multi-region multi-modal radiomics features, volume features, as well as clinical features led to the best prediction performance. The radiomics_clinical model is able to stratify patients into three groups, which helps clinical decision-making and selecting patients for (de-)escalation trials and/or adjuvant treatment.

Researchers have studied the fairness of algorithms to ensure that the AI algorithms were equitable and fair. Newton et al.’s study [50] revealed a correlation between a model’s accuracy and an individual’s skin tone classification due to the underrepresentation of individuals with darker skin in benchmark datasets. Statistical analysis of the segmentation result with respect to various subgroups revealed that the performance of the segmentation model was not affected by age and gender. For patients who did not receive chemotherapy, the ${\mathrm{DSC}}_{\mathrm{agg}}$ is lower, and sensitivity is higher than overall performance. This means that segmentation quality is lower for such patients, but the algorithm is able to detect them with a DSC threshold of 0.1. The segmentation performance for HPV-positive patients was lower and for HPV-negative patients was higher than the overall performance. The segmentation performance is impacted by variations in texture features and morphological characteristics. AK Tahari et al. conducted a study to evaluate the differences in morphological features in primary tumours and regional lymph nodes between HPV-positive and HPV-negative patients. The findings revealed that HPV-negative patients had larger primary tumours, higher heterogeneity, and a higher standardized uptake value [51]. Furthermore, Fujita et al. compared texture features between HPV-positive and HPV-negative non-OPC patients on CT scans and found that numerous texture analysis features demonstrated statistically significant differences between the two groups [52]. These findings highlight the need to consider HPV status in the texture analysis and segmentation of head and neck cancer. Furthermore, small tumours and lymph nodes had lower performance, and large tumours and lymph nodes had higher segmentation performance. This could be explained due to the fact that the non-overlapping of a few pixels for small tumours had a significant impact on the DSC and large tumours are generally easier to segment. While the size of the lesion had an impact on the segmentation performance, there was no statistically significant difference in sensitivity. It is crucial to inform the end users whether the segmentation model was influenced by the presence or absence of clinical features. The statistical analysis of the radiomics_clinical survival prediction model for several subcohorts revealed that performance was not impacted except for those who have not received chemotherapy or are HPV-negative, as well as for large tumour size. For these three subgroups, the performance of the model was significantly lower than the overall performance, indicating that the survival prediction model should be used with caution in certain cases.

Additionally, a SHAP analysis was conducted to better understand the contributions of each feature to the prediction. The global SHAP analysis highlighted glszm_GrayLevelNonUniformity from lymph nodes in PET, firstorder_Range from tumours in CT, and glszm_largeAreaLowGrayLevelEmphasis from lymph nodes in PET as the most important radiomics features. This result emphasises the significance of texture features and aligns with previous findings that tumour features in CT and lymph node features in PET are more informative. The SHAP analysis also revealed that HPV infection was associated with a favourable prognosis and consistent with other studies that show HPV-related H&N cancers are often found in younger, healthier patients with high economic status and high-risk sexual behaviour, leading to improved prognosis [53].

There were a few limitations in this study. We observed that the performance of the segmentation model and survival prediction model was impacted by chemotherapy and HPV status. There is a need to investigate bias mitigation strategies in the future to see if we can ensure that the algorithm is unbiased with respect to these identified biases. We have limited data on radiation type and chemotherapy dosage to investigate its impact on the model performance. The non-availability of the clinical data such as HPV status can hamper the fairness analysis of the segmentation and survival prediction models. While we have extensively validated our segmentation and survival prediction model on unseen datasets, there is still a need for prospective validation to study the usability and clinical utility of the models. There is a need to quantitatively study uncertainty estimation with inter-observer variability to further instil confidence in the confidence scores. There is also a need to evaluate the explainability of the proposed clinical_radiomics model in clinical practice.

In this paper, we proposed an end-to-end framework for tumours and lymph detection, segmentation, and prediction. We increase the reliability of the segmentations by identifying silent failures and reducing false positives by using uncertainty estimates. We demonstrated by qualitative and quantitative analysis that the uncertainty maps can identify salient failures and can also aid in false positive reduction. We also explored the prognostic potential of radiomics features extracted from tumours and lymph nodes in PET and CT. We also performed a quantitative analysis of the segmentation and survival prediction models to ensure fairness and equitable performance with respect to age, gender, chemotherapy status, HPV status, and lesion size. We aimed to draw the attention of healthcare professionals to the performance outliers of the models. Furthermore, we employed SHAP analysis for the interpretability of the model and correlated the insights with clinical knowledge. In future, prospective in silico studies that evaluate the performance of the clinicians with the aid of these segmentation and survival prediction models as well as quantify the usefulness of uncertainty estimation and explainability need to be carried out. Furthermore, fair models that mitigate the identified biases may also be developed in the future.

## 5. Conclusions

An end-to-end segmentation and recurrence-free survival prediction framework has been proposed that incorporates uncertainty estimation, fairness, and explainability. The uncertainty estimates have been used to identify failure cases and for per image false positive reduction. The recurrence-free survival prediction model can be used to stratify patients into distinct risk groups for treatment (de-)escalation trials and clinical decision support. The fairness of models has been evaluated to identify biases and inform the end-users of performance outliers.

## Figures and Tables

**Table 1:** Clinical characteristics of patients included in this study.

Characteristic	Training Set (n = 524)	Test Set (n = 359)	*p*-Value
**Gender (M/F)**	429/95	296/63	0.825
**Age (years)**	61.0 (54.0–67.0)	59.0 (53.2–66.0)	0.024
**Weight (kg)**	80.0 (67.8–92.1)	81.0 (66.0–96.0)	0.570
**Chemotherapy (Yes/No)**	457/67	311/48	0.800
**Tobacco (Yes/No/NA)**	108/111/305	97/94/168	-
**Alcohol (Yes/No/NA)**	112/70/342	103/45/211	-
**Surgery (Yes/No/NA)**	51/255/218	34/325/0	-
**HPV status (+/−/NA)**	279/61/184	203/20/136	-
**Performance status (0/1/2/3/4/NA)**	91/137/11/3/1/281	116/96/21/5/3/118	-

**Table 2:** Fairness evaluation of Dice score coefficients (DSC), aggregated Dice (DSCagg), and sensitivity metrics for primary tumour and lymph nodes during fivefold cross-validation.

		N (%)	*DSC* Tumour	*DSC* Lymph	$DSC_{agg}$ GTVp	$DSC_{agg}$ GTVn	Sensitivity Tumour	Sensitivity Lymph
Total (All)		488	0.725	0.653	0.808	0.780	0.964	0.878
Age	0–50	44 (9.02%)	0.745	0.629	0.820	0.780	0.943	0.824
	50–60	178 (36.48%)	0.715	0.671	0.796	0.786	0.963	0.888
	60–70	172 (35.3%)	0.737	0.649	0.822	0.770	0.962	0.897
	>70	94 (19.26%)	0.710	0.639	0.794	0.784	0.979	0.854
Gender	Male	402 (82.4%)	0.726	0.663	0.809	0.782	0.966	0.874
	Female	86 (17.6%)	0.719	0.606	0.803	0.758	0.953	0.899
Chemotherapy	Yes	422 (86.5%)	0.741	0.669	0.820	0.781	0.947	0.918
	No	66 (13.5%)	0.623	0.551	0.627 *	0.756	0.967 *	0.873
HPV	Yes	43 (8.81%)	0.715	0.701	0.797	0.789	0.988	0.852 *
	No	274 (56.15%)	0.788	0.563	0.830	0.758	0.962	0.904 *
	NA	171 (35.04%)	0.724	0.598	0.817	0.764	0.964	0.846
Tumour Size (cm^3^)	Small = 0–4.03	122 (25%)	0.558 *	-	0.552 *	-	0.939	0.857
	Medium = 4.03–7.70	122 (25%)	0.755	-	0.756 *	-	0.996	0.924
	Large = 7.70–17.03	122 (25%)	0.750	-	0.774	-	0.963	0.874
	Extra Large =17.3–184.5	122 (25%)	0.836 *	-	0.854 *	-	0.959	0.860
Lymph Node Size (cm^3^)	Small = 0–2.90	122 (25%)	-	0.381 *	-	0.322 *	0.930	0.889
	Medium = 2.90–11.8	122 (25%)	-	0.652 *	-	0.680 *	0.988	0.859
	Large = 11.8–24.0	122 (25%)	-	0.768	-	0.782	0.971	0.865
	Extra Large = 24.0–124.5	122 (25%)	-	0.811 *	-	0.812 *	0.967	0.901

* shows that there is a statistically significant difference between the subgroup and total metrics.

**Table 3:** The C-index results on fivefold cross-validation and test set.

No.	Method	Fivefold Cross-Validation
1	CT_all	0.659 ± 0.063
2	CT_only_tumour	0.627 ± 0.062
3	CT_largest_tumour	0.587 ± 0.040
4	CT_only_lymph_node	0.608 ± 0.059
5	CT_largest_lymph_node	0.605 ± 0.060
6	PET_all	0.622 ± 0.077
7	PET_only_tumour	0.603 ± 0.065
8	PET_largest_tumour	0.569 ± 0.054
9	PET_only_lymph_node	0.635 ± 0.074
10	PET_largest_lymph_node	0.644 ± 0.084
11	volume	0.598 ± 0.051
12	clinical	0.552 ± 0.032
13	radiomics_clinical	0.682 ± 0.083
**No.**	**Method**	**Test Set**
1	radiomics_clinical	0.672

**Table 4:** Fairness of radiomics survival prediction with different subgroups of age, gender, chemotherapy, HPV status, and lesion size.

Characteristics	Group	Number (Percentage)	C-Index	*p*-Value
Total (All)	-	488 (100%)	0.682	-
Age	0–50	44 (9.0%)	0.846	0.126
	50–60	178 (36.5%)	0.609	0.217
	60–70	172 (35.2%)	0.704	0.368
	>70	94 (19.3%)	0.728	0.352
Gender	Male	402 (82.4%)	0.663	0.325
	Female	86 (17.6%)	0.759	0.147
Chemotherapy	Yes	422 (86.5%)	0.685	0.408
	No	66 (13.5%)	0.555	0.022 *
HPV	Yes	43 (8.81%)	0.650	0.451
	No	274 (56.15%)	0.551	0.006 *
	N/A	171 (35.0)	0.709	0.321
Tumour size (cm^3^)	Small = 0–4.03	122 (25%)	0.654	0.271
	Medium = 4.03–7.70	122 (25%)	0.737	0.363
	Large = 7.70–17.3	122 (25%)	0.561	0.037 *
	Extra Large = 17.3–184.5	122 (25%)	0.633	0.378
Lymph node size (cm^3^)	Small = 0–2.90	122 (25%)	0.606	0.134
	Medium = 2.90–11.8	122 (25%)	0.664	0.327
	Large = 11.8–24.0	122 (25%)	0.847	0.053
	Extra Large = 24.0–124.5	122 (25%)	0.617	0.224

* shows that there is a statistically significant difference for the subgroup when compared with overall population.

## Data Availability

The data can be obtained after signing the end user agreement as a part of Hecktor 2022 challenge at MICCAI 2022 available at: https://hecktor.grand-challenge.org/participants/registration/create/ (accessed on 20 March 2023).

## Appendix A

**Table A1:** Description on the type of radiomics features that are extracted from each region of interest from PET and CT images. More information on the type of features is present in PyRadiomics documentation (https://pyradiomics.readthedocs.io/en/latest/features.html (accessed on 20 March 2023)).

Radiomics Feature	Description
Gray level co-occurrence matrix (GLCM)	Gray level co-occurrence matrix features describe the second order joint probability function of the voxel intensities. These features include measures such as contrast, correlation, energy, and homogeneity. In this study, 24 GLCM features were extracted using PyRadiomics.
Gray level difference matrix (GLDM)	Gray level difference matrix features describe the distribution of gray level differences within the ROI. These features include measures such as coarseness, contrast, and busyness. In this study, 14 GLDM features were extracted using PyRadiomics.
Gray level run length matrix (GLRLM)	Gray level run length matrix features describe the length of runs of consecutive voxels with the same gray level. These features include measures such as short-run emphasis, long-run emphasis, and run percentage. In this study, 16 GLRLM features were extracted using PyRadiomics.
Gray level size zone matrix (GLSZM)	Gray level size zone matrix features describe the size of zones of consecutive voxels with the same gray level. These features include measures such as zone size, zone percentage, and zone entropy. In this study, 16 GLSZM features were extracted using PyRadiomics.
Neighbouring gray tone difference matrix (NGTDM)	Neighbouring gray tone difference matrix features describe the distribution of voxel-level texture primitive patterns. These features include measures such as coarseness and contrast. In this study, 5 NGTDM features were extracted using PyRadiomics.
First order statistics	First order statistics describe the distribution of voxel intensities within the ROI. These features include measures such as mean, median, skewness, and kurtosis. In this study, 18 first order features were extracted using PyRadiomics.
Shape-based (3D)	Shape features describe the shape and size of the ROI. These features include measures such as volume, surface area, sphericity, and compactness. In this study, 14 shape features were extracted using PyRadiomics.

**Table A2:** Description of the radiomics features used in the proposed radiomics_clinical model for recurrence-free survival prediction.

Class	Radiomics Feature	Definition
**First order:** First order statistics describe the distribution of voxel intensities within the image region defined by the mask through commonly used and basic metrics.	Original_firstorder_Range	The range of first order value in the ROI
	Original_firstorder_10Percentile	The 10th percentile of first order value.
	Original_firstorder_ TotalEnergy	Total Energy is the value of the Energy feature scaled by the volume of the voxel in cubic mm
	Original_firstorder_ InterquartileRange	The different value 25th and 75th percentile of the first order value.
**GLSZM:** A gray level size Zone (GLSZM) quantifies gray level zones in an image	original_glszm_ grayLevelNonUniformity	GrayLevelNonUniformity measures the variability of gray-level intensity values in the GLSZM array.
	Original_glszm_ LargeAreaLowGrayLevelEmphasis	It measures the proportion in the image of the joint distribution of larger size zones with lower gray level values
	Original_glszm_ SizeZoneNonUniformity	It measures the variability of size zone volumes throughout the GLSZM array, with a lower value indicating more homogeneity among zone size volumes in the GLSZM array.
	Original_glszm_ ZoneEntropy	It measures the uncertainty/randomness in the distribution of zone sizes and GLSZM levels
**GLRLM:** A gray level run length matrix (GLRLM) quantifies gray level runs, which are defined as the length in number of pixels, of consecutive pixels that have the same gray level value.	Original_glrlm_ GrayLevelNonUniformity	It measures the similarity of gray level intensity values in the GLRLM array.
	Original_glrlm_ RunLengthNonUniformity	It measures the similarity of run lengths throughout the GLRLM array, with a lower value indicating more homogeneity among run lengths in the GLRLM array
**GLDM:** A gray level dependence matrix (GLDM) quantifies gray level dependencies in an image	Original_gldm_ SmallDependenceEmphasis	A measure of the distribution of large dependencies, with a greater value indicative of larger dependence and more homogeneous textures.

## References

[1] Gormley M., Creaney G., Schache A., Ingarfield K., Conway D.I. (2022). Reviewing the Epidemiology of Head and Neck Cancer: Definitions, Trends and Risk Factors. Br. Dent. J..

[2] Warnakulasuriya S. (2009). Global Epidemiology of Oral and Oropharyngeal Cancer. Oral Oncol..

[3] Marcus C., Sheikhbahaei S., Shivamurthy V.K.N., Avey G., Subramaniam R.M. (2021). PET Imaging for Head and Neck Cancers. Radiol. Clin. N. Am..

[4] Rygalski C.J., Zhao S., Eskander A., Zhan K.Y., Mroz E.A., Brock G., Silverman D.A., Blakaj D., Bonomi M.R., Carrau R.L. (2021). Time to Surgery and Survival in Head and Neck Cancer. Ann. Surg. Oncol..

[5] Mehanna H., Wong W.-L., McConkey C.C., Rahman J.K., Robinson M., Hartley A.G.J., Nutting C., Powell N., Al-Booz H., Robinson M. (2016). PET-CT Surveillance versus Neck Dissection in Advanced Head and Neck Cancer. N. Engl. J. Med..

[6] Escott E.J. (2013). Role of Positron Emission Tomography/Computed Tomography (PET/CT) in Head and Neck Cancer. Radiol. Clin. N. Am..

[7] Lonneux M., Hamoir M., Reychler H., Maingon P., Duvillard C., Calais G., Bridji B., Digue L., Toubeau M., Grégoire V. (2010). Positron Emission Tomography with [18F]fluorodeoxyglucose Improves Staging and Patient Management in Patients with Head and Neck Squamous Cell Carcinoma: A Multicenter Prospective Study. J. Clin. Oncol..

[8] van der Veen J., Gulyban A., Nuyts S. (2019). Interobserver Variability in Delineation of Target Volumes in Head and Neck Cancer. Radiother. Oncol..

[9] Du G., Cao X., Liang J., Chen X., Zhan Y. (2020). Medical Image Segmentation Based on U-Net: A Review. J. Imaging Sci. Technol..

[10] Siddique N., Paheding S., Elkin C.P., Devabhaktuni V. (2021). U-Net and Its Variants for Medical Image Segmentation: A Review of Theory and Applications. IEEE Access.

[11] Çiçek Ö., Abdulkadir A., Lienkamp S.S., Brox T., Ronneberger O. 3D U-Net: Learning Dense Volumetric Segmentation from Sparse Annotation. Proceedings of the Medical Image Computing and Computer-Assisted Intervention—MICCAI 2016.

[12] Iantsen A., Visvikis D., Hatt M. (2021). Squeeze-and-Excitation Normalization for Automated Delineation of Head and Neck Primary Tumors in Combined PET and CT Images. Head and Neck Tumor Segmentation.

[13] Isensee F., Jaeger P.F., Kohl S.A.A., Petersen J., Maier-Hein K.H. (2021). nnU-Net: A Self-Configuring Method for Deep Learning-Based Biomedical Image Segmentation. Nat. Methods.

[14] Jungo A., Reyes M. (2019). Assessing Reliability and Challenges of Uncertainty Estimations for Medical Image Segmentation. Lecture Notes in Computer Science.

[15] Zhao Y., Yang C., Schweidtmann A., Tao Q. (2022). Efficient Bayesian Uncertainty Estimation for nnU-Net. Proceedings of the Medical Image Computing and Computer Assisted Intervention—MICCAI 2022.

[16] Czolbe S., Arnavaz K., Krause O., Feragen A. (2021). Is Segmentation Uncertainty Useful. Lecture Notes in Computer Science.

[17] Lambin P., Leijenaar R.T.H., Deist T.M., Peerlings J., de Jong E.E.C., van Timmeren J., Sanduleanu S., Larue R.T.H.M., Even A.J.G., Jochems A. (2017). Radiomics: The Bridge between Medical Imaging and Personalized Medicine. Nat. Rev. Clin. Oncol..

[18] Refaee T., Salahuddin Z., Frix A.-N., Yan C., Wu G., Woodruff H.C., Gietema H., Meunier P., Louis R., Guiot J. (2022). Diagnosis of Idiopathic Pulmonary Fibrosis in High-Resolution Computed Tomography Scans Using a Combination of Handcrafted Radiomics and Deep Learning. Front. Med..

[19] Aerts H.J.W.L., Velazquez E.R., Leijenaar R.T.H., Parmar C., Grossmann P., Carvalho S., Bussink J., Monshouwer R., Haibe-Kains B., Rietveld D. (2014). Decoding Tumour Phenotype by Noninvasive Imaging Using a Quantitative Radiomics Approach. Nat. Commun..

[20] Kanchan G., Chen Q., Feng X. (2022). Head and neck tumor segmentation with deeply-supervised 3D UNet and progression-free survival prediction with linear model. Head and Neck Tumor Segmentation and Outcome Prediction: Second Challenge, HECKTOR 2021, Held in Conjunction with MICCAI 2021, Strasbourg, France, 27 September 2021.

[21] Martinez-Larraz A., Asenjo J.M., Rodríguez B.Á. (2022). PET/CT head and neck tumor segmentation and progression free survival prediction using deep and machine learning techniques. Head and Neck Tumor Segmentation and Outcome Prediction: Second Challenge, HECKTOR 2021, Held in Conjunction with MICCAI 2021, Strasbourg, France, 27 September 2021.

[22] Fatan M., Hosseinzadeh M., Askari D., Sheikhi H., Rezaeijo S.M., Salmanpour M.R. (2022). Fusion-based head and neck tumor segmentation and survival prediction using robust deep learning techniques and advanced hybrid machine learning systems. Head and Neck Tumor Segmentation and Outcome Prediction: Second Challenge, HECKTOR 2021, Held in Conjunction with MICCAI 2021, Strasbourg, France, 27 September 2021.

[23] Keek S.A., Wesseling F.W.R., Woodruff H.C., van Timmeren J.E., Nauta I.H., Hoffmann T.K., Cavalieri S., Calareso G., Primakov S., Leijenaar R.T.H. (2021). A Prospectively Validated Prognostic Model for Patients with Locally Advanced Squamous Cell Carcinoma of the Head and Neck Based on Radiomics of Computed Tomography Images. Cancers.

[24] Mukherjee P., Cintra M., Huang C., Zhou M., Zhu S., Colevas A.D., Fischbein N., Gevaert O. (2020). CT-Based Radiomic Signatures for Predicting Histopathologic Features in Head and Neck Squamous Cell Carcinoma. Radiol Imaging Cancer.

[25] Xie C., Yang P., Zhang X., Xu L., Wang X., Li X., Zhang L., Xie R., Yang L., Jing Z. (2019). Sub-Region Based Radiomics Analysis for Survival Prediction in Oesophageal Tumours Treated by Definitive Concurrent Chemoradiotherapy. EBioMedicine.

[26] Bogowicz M., Riesterer O., Stark L.S., Studer G., Unkelbach J., Guckenberger M., Tanadini-Lang S. (2017). Comparison of PET and CT Radiomics for Prediction of Local Tumor Control in Head and Neck Squamous Cell Carcinoma. Acta Oncol..

[27] Lv W., Feng H., Du D., Ma J., Lu L. (2021). Complementary Value of Intra- and Peri-Tumoral PET/CT Radiomics for Outcome Prediction in Head and Neck Cancer. IEEE Access.

[28] McCartney G., Popham F., McMaster R., Cumbers A. (2019). Defining Health and Health Inequalities. Public Health.

[29] Paulus J.K., Kent D.M. (2020). Predictably Unequal: Understanding and Addressing Concerns That Algorithmic Clinical Prediction May Increase Health Disparities. NPJ Digit Med..

[30] Lekadir K., Osuala R., Gallin C., Lazrak N., Kushibar K., Tsakou G., Aussó S., Alberich L.C., Marias K., Tsiknakis M. (2021). FUTURE-AI: Guiding Principles and Consensus Recommendations for Trustworthy Artificial Intelligence in Medical Imaging. arXiv.

[31] Seyyed-Kalantari L., Liu G., McDermott M., Chen I.Y., Ghassemi M. (2021). CheXclusion: Fairness Gaps in Deep Chest X-Ray Classifiers. Pac. Symp. Biocomput..

[32] Puyol-Antón E., Ruijsink B., Piechnik S.K., Neubauer S., Petersen S.E., Razavi R., King A.P. Fairness in Cardiac MR Image Analysis: An Investigation of Bias Due to Data Imbalance in Deep Learning Based Segmentation. Proceedings of the Medical Image Computing and Computer Assisted Intervention—MICCAI 2021.

[33] Andrearczyk V., Oreiller V., Abobakr M., Akhavanallaf A., Balermpas P., Boughdad S., Capriotti L., Castelli J., Le Rest C.C., Decazes P., Andrearczyk V., Oreiller V., Hatt M., Depeursinge A. (2022). Overview of the HECKTOR Challenge at MICCAI 2022: Automatic Head and Neck Tumor Segmentation and Outcome Prediction in PET/CT. Head and Neck Tumor Segmentation and Outcome Prediction.

[34] Oreiller V., Andrearczyk V., Jreige M., Boughdad S., Elhalawani H., Castelli J., Vallières M., Zhu S., Xie J., Peng Y. (2022). Head and Neck Tumor Segmentation in PET/CT: The HECKTOR Challenge. Med. Image Anal..

[35] Isensee F., Jäger P.F., Full P.M., Vollmuth P., Maier-Hein K.H. (2021). nnU-Net for Brain Tumor Segmentation. Proceedings of the Brainlesion: Glioma, Multiple Sclerosis, Stroke and Traumatic Brain Injuries.

[36] He K., Zhang X., Ren S., Sun J. (2016). Identity Mappings in Deep Residual Networks. arXiv.

[37] Hu J., Shen L., Albanie S., Sun G., Wu E. (2020). Squeeze-and-Excitation Networks. IEEE Trans. Pattern Anal. Mach. Intell..

[38] Schlemper J., Oktay O., Schaap M., Heinrich M., Kainz B., Glocker B., Rueckert D. (2019). Attention Gated Networks: Learning to Leverage Salient Regions in Medical Images. Med. Image Anal..

[39] Sudre C.H., Li W., Vercauteren T., Ourselin S., Jorge Cardoso M. (2017). Generalised Dice Overlap as a Deep Learning Loss Function for Highly Unbalanced Segmentations. Deep Learn Med Image Anal Multimodal Learn Clin Decis Support.

[40] Tixier F., Le Rest C.C., Hatt M., Albarghach N., Pradier O., Metges J.-P., Corcos L., Visvikis D. (2011). Intratumor Heterogeneity Characterized by Textural Features on Baseline 18F-FDG PET Images Predicts Response to Concomitant Radiochemotherapy in Esophageal Cancer. J. Nucl. Med..

[41] Whang S.E., Lee J.-G. (2020). Data Collection and Quality Challenges for Deep Learning. VLDB J..

[42] Alves N., Schuurmans M., Litjens G., Bosma J.S., Hermans J., Huisman H. (2022). Fully Automatic Deep Learning Framework for Pancreatic Ductal Adenocarcinoma Detection on Computed Tomography. Cancers.

[43] McKinney S.M., Sieniek M., Godbole V., Godwin J., Antropova N., Ashrafian H., Back T., Chesus M., Corrado G.S., Darzi A. (2020). International Evaluation of an AI System for Breast Cancer Screening. Nature.

[44] Saha A., Hosseinzadeh M., Huisman H. (2021). End-to-End Prostate Cancer Detection in bpMRI via 3D CNNs: Effects of Attention Mechanisms, Clinical Priori and Decoupled False Positive Reduction. Med. Image Anal..

[45] Meng M., Peng Y., Bi L., Kim J. (2022). Multi-Task Deep Learning for Joint Tumor Segmentation and Outcome Prediction in Head and Neck Cancer. Lecture Notes in Computer Science.

[46] Rebaud L., Escobar T., Khalid F., Girum K., Buvat I. (2022). Simplicity Is All You Need: Out-of-the-Box nnUNet Followed by Binary-Weighted Radiomic Model for Segmentation and Outcome Prediction in Head and Neck PET/CT.

[47] Wang K., Li Y., Dohopolski M., Peng T., Lu W., Zhang Y., Wang J. (2022). Recurrence-Free Survival Prediction under the Guidance of Automatic Gross Tumor Volume Segmentation for Head and Neck Cancers. arXiv.

[48] Carvalho S., Leijenaar R.T.H., Troost E.G.C., van Timmeren J.E., Oberije C., van Elmpt W., de Geus-Oei L.-F., Bussink J., Lambin P. (2018). 18F-Fluorodeoxyglucose Positron-Emission Tomography (FDG-PET)-Radiomics of Metastatic Lymph Nodes and Primary Tumor in Non-Small Cell Lung Cancer (NSCLC)—A Prospective Externally Validated Study. PLoS ONE.

[49] Kim J.H., Choi K.Y., Lee S.-H., Lee D.J., Park B.J., Yoon D.Y., Rho Y.-S. (2020). The Value of CT, MRI, and PET-CT in Detecting Retropharyngeal Lymph Node Metastasis of Head and Neck Squamous Cell Carcinoma. BMC Med. Imaging.

[50] Kinyanjui N.M., Odonga T., Cintas C., Codella N.C.F., Panda R., Sattigeri P., Varshney K.R. (2020). Fairness of classifiers across skin tones in dermatology. Medical Image Computing and Computer Assisted Intervention–MICCAI 2020: 23rd International Conference, Lima, Peru, 4–8 October 2020.

[51] Tahari A.K., Alluri K.C., Quon H., Koch W., Wahl R.L., Subramaniam R.M. (2014). FDG PET/CT imaging of Oropharyngeal SCC: Characteristics of HPV positive and negative tumors. Clin. Nucl. Med..

[52] Fujita A., Buch K., Li B., Kawashima Y., Qureshi M.M., Sakai O. (2016). Difference between HPV-positive and HPV-negative non-oropharyngeal head and neck cancer: Texture analysis features on CT. J. Comput. Assist. Tomogr..

[53] Economopoulou P., Kotsantis I., Psyrri A. (2020). Special Issue about Head and Neck Cancers: HPV Positive Cancers. Int. J. Mol. Sci..

